# Enrollment of Diverse Populations in the INGENIOUS Pharmacogenetics Clinical Trial

**DOI:** 10.3389/fgene.2020.00571

**Published:** 2020-06-25

**Authors:** Ebony Shah-Williams, Kenneth D. Levy, Yong Zang, Ann M. Holmes, Christa Stoughton, Paul Dexter, Todd C. Skaar

**Affiliations:** ^1^Department of Medical and Molecular Genetics, Indiana University, Indianapolis, IN, United States; ^2^Department of Medicine, Indiana University, Indianapolis, IN, United States; ^3^Department of Biostatistics, Indiana University, Indianapolis, IN, United States; ^4^Department of Health Policy and Management, Fairbanks School of Public Health, Indiana University–Purdue University Indianapolis, Indianapolis, IN, United States; ^5^Department of Urology, Indiana University Hospital, Indianapolis, IN, United States; ^6^Regenstrief Institute for Health Care, Indianapolis, IN, United States

**Keywords:** diverse, clinical trial, African-American, INGENIOUS, enrollment, pharmacogenomics, MUA/P

## Abstract

Recruitment of diverse populations and subjects living in Medically Underserved Areas and Populations (MUA/P’s) into clinical trials is a considerable challenge. Likewise, representation of African-Americans in pharmacogenetic trials is often inadequate, but critical for identifying genetic variation within and between populations. To identify enrollment patterns and variables that predict enrollment in a diverse underserved population, we analyzed data from the INGENIOUS (Indiana GENomics Implementation and Opportunity for the UnderServed), pharmacogenomics implementation clinical trial conducted at a community hospital for underserved subjects (Safety net hospital), and a statewide healthcare system (Academic hospital). We used a logistic regression model to identify patient variables that predicted successful enrollment after subjects were contacted and evaluated the reasons that clinical trial eligible subjects refused enrollment. In both healthcare systems, African-Americans were less likely to refuse the study than non-Hispanic Whites (Safety net, OR = 0.68, and *p* < 0.002; Academic hospital, OR = 0.64, and *p* < 0.001). At the Safety net hospital, other minorities were more likely to refuse the study than non-Hispanic Whites (OR = 1.58, *p* < 0.04). The odds of refusing the study once contacted increased with patient age (Safety net hospital, OR = 1.02, *p* < 0.001, Academic hospital, OR = 1.02, and *p* < 0.001). At the Academic hospital, females were less likely to refuse the study than males (OR = 0.81, *p* = 0.01) and those not living in MUA/P’s were less likely to refuse the study than those living in MUA/P’s (OR = 0.81, *p* = 0.007). The most frequent barriers to enrollment included not being interested, being too busy, transportation, and illness. A lack of trust was reported less frequently. In conclusion, African-Americans can be readily recruited to pharmacogenetic clinical trials once contact has been successfully initiated. However, health care initiatives and increased recruitment efforts of subjects living in MUA/Ps are needed. Enrollment could be further enhanced by improving research awareness and knowledge of clinical trials, reducing time needed for participation, and compensating for travel.

## Introduction

Randomized clinical trials are the gold standard for testing the safety and efficacy of therapeutic products, medical devices and clinical procedures (Minneci and Deans, 2018). In some cases, subjects may benefit from participating in clinical trials, which offer better alternatives in treatment compared to the standard of care for certain diseases and are provided with extra support and monitoring from health care providers. However, African-Americans, who are disproportionally diagnosed with higher rates of various diseases such as hypertension, diabetes, and stroke, compared to non-Hispanic Whites, are historically underrepresented in clinical trials (Thorpe et al., 2016; Musemwa and Gadegbeku, 2017; No author list, 2018). This creates racial disparities in receiving benefits from clinical trials. It also often limits the ability of researchers to extrapolate the findings and use pharmacogenomics to personalize drug therapies across races (Perera et al., 2014; Shendre et al., 2018). Additionally, racial disparities exist in the incidence of adverse drug events, making minority populations particularly important to include in pharmacogenetic clinical trials (Moffett et al., 2013; Chalasani et al., 2017).

A variety of strategies have been reported in an attempt to lessen health disparities in minority populations across many diseases (Smith et al., 2016). One of these strategies is to increase community involvement at the beginning and throughout the research process (The National Academies of Sciences Engineering, and Medicine, 2016). Thus, many studies have focused on barriers that impede African-Americans from enrolling into clinical trials. Several of them have focused on health inequities and medical practices that have historically prevented African-Americans from enrolling into clinical trials (Lincoln et al., 2018). However, barriers to enrollment in pharmacogenetic trials may be different. For example, diverse populations may be more willing to participate in pharmacogenetic trials because identifying genetic contributions to reduced efficacy could provide minority populations valuable explanations for the reduced efficacy that does not blame it on their lifestyles. On the other hand, Lee et al. (2018) have reported factors that impact pharmacogenetic testing in primarily minority populations, including concerns about the impact of the test results, reliability of test, and concerns about privacy. In an effort to understand the impact of pharmacogenetic testing in minority and underserved populations, we have recently concluded enrollment to our INdiana GENomics Implementation: an Opportunity for the UnderServed (INGENIOUS) clinical trial (Eadon et al., 2016).

Herein, we report the rates of enrollment of minority and underserved populations along with reasons for refusing to enroll. This study provides a unique opportunity to report enrollment patterns in a diverse population from a large safety net and a state-wide health care system, which include a large portion of subjects who live in Medically Underserved Areas and Populations (MUA/P’s), and/or are an African-American patient population. The aim of this analysis is to (1) determine differences in enrollment patterns in diverse populations and (2) evaluate patient variables that may predict enrollment into a pharmacogenetic clinical trial and (3) evaluate reasons clinical trial eligible subjects did not enroll in the INGENIOUS trial.

## Materials and Methods

The INGENIOUS trial (ClinicalTrials.gov Identifier: NCT02297126) was a prospective, randomized controlled pharmacogenetics clinical trial. The study enrolled subjects to evaluate clinical responses and economic impact of implementing a pharmacogenetics program at a safety-net urban hospital, and an Academic hospital across the state of Indiana. The study design was previously described (Eadon et al., 2016). INGENIOUS data were analyzed from subjects who were contacted between January 2017 and April 2018, using data collected by the research assistants, who contacted the eligible subjects.

### Study Selection and Patient Demographics

#### Patient Inclusion/Exclusion Criteria

**FIGURE 1:**
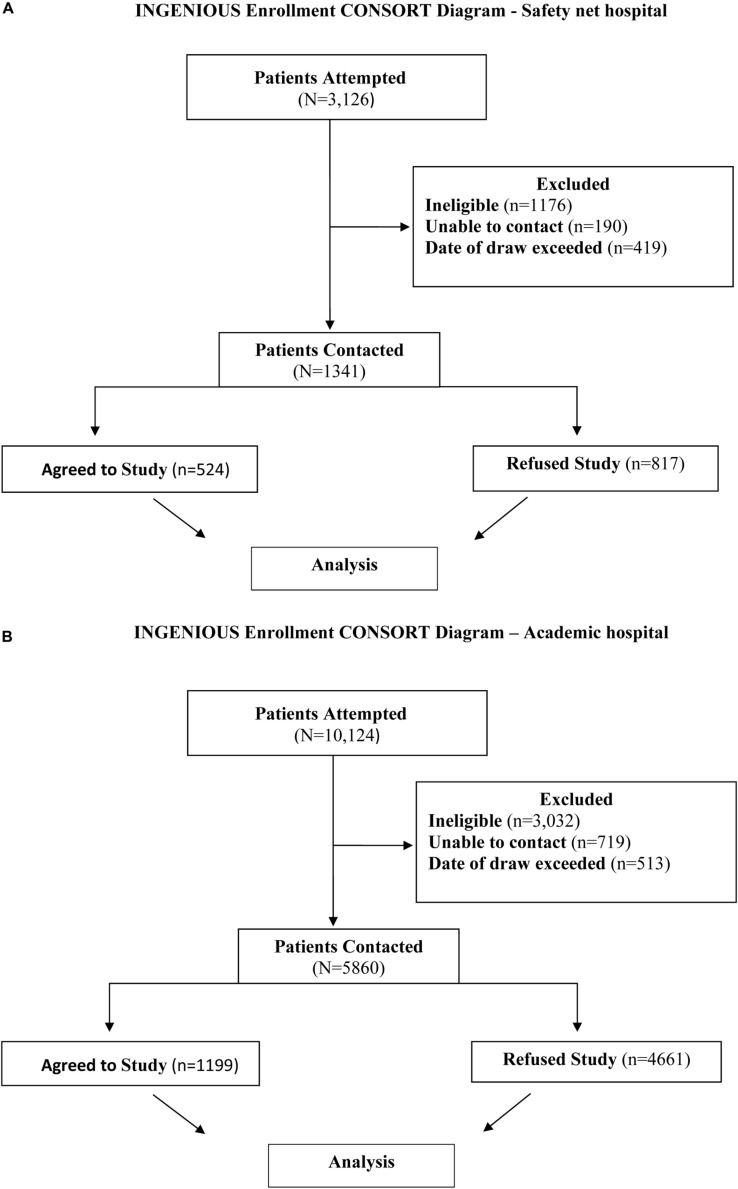
**(A)** INGENIOUS CONSORT Diagram for the inclusion of data analyzed at the Safety net hospital. The final analysis included 1341 subjects, 524 who agreed to the study and 817 subjects who refused the study. **(B)** INGENIOUS CONSORT Diagram for the inclusion of data analyzed at the Academic hospital. The final analysis included 1199 subjects who agreed to the study and 4661 subjects who refused the study.

The inclusion criteria included: (1) ability to consent, (2) prescribed 1 of the 27 index medications, (3) must not have been prescribed the index medication in the past 13 months, and (4) be at least 18 years of age. Exclusion criteria included: (1) not English speaking, (2) subjects previously genotyped. Subjects were randomized into control and intervention arms. Subjects randomized to the control arm received standard of care, were not genotyped, were not contacted, and outcomes data for the trial endpoint analyses were obtained from the medical records. Subjects randomized to the intervention arm (pharmaco-genotyping) were actively recruited and consented via telephone or in clinics. Subjects in the intervention arm who were not successfully contacted for recruitment or were unable to complete the enrollment process within 5 days of receiving the targeted medication were excluded from this analysis (Figure 1). Subjects who successfully enrolled and were consented received compensation of $50. At both hospital systems, hypothesis testing, criteria for inclusion/exclusion, data collection, and analysis were identical, but varied in patient demographics.

#### Medically Underserved Areas and Population

Medically Underserved Areas and Populations are designated geographic areas and populations with a paucity of key health care services as assessed by the Health Resource & Service Administration (HRSA). Subjects identified living in MUA/Ps are based on the Index of Medical Underservice (IMU). The four criteria for assigning MUA/P designations are population-to provider ratio, percent of the population below the federal poverty level, percent population over the age of 65, and the percent infant mortality rate^1^.

#### Patient Selection at the Safety net Hospital

**FIGURE 2:**
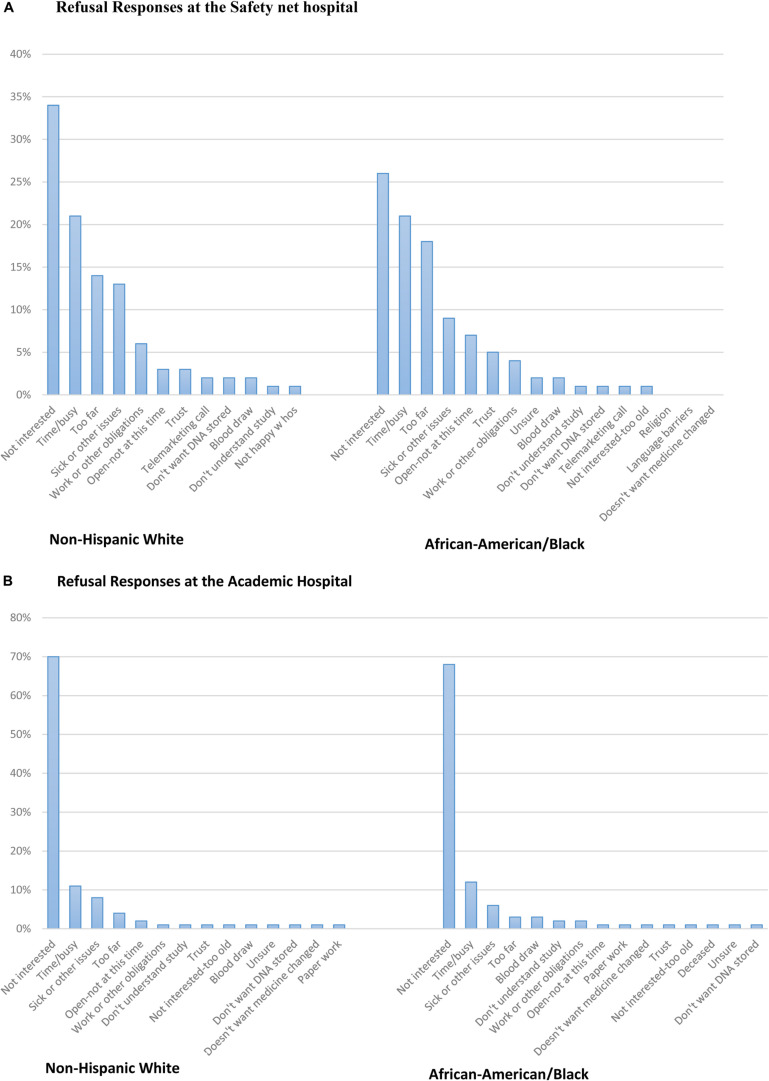
**(A)** Refusal responses at the Safety net hospital. **(B)** Refusal responses at the Academic hospital.

3,126 subjects with recruitment enrollment data were identified at the Safety net hospital and extracted from the REDCap database (Harris et al., 2009). A total of 1,785 subjects were excluded from the study for the following reasons: 1,176 subjects were ineligible based on enrollment criteria, 190 subjects were unable to be contacted, and 419 subjects did not complete the enrollment process within 5 days from receiving the prescription for the targeted medication. After exclusions, there were 1,341 eligible subjects successfully contacted and included in the final analysis (Figures 1A, 2A). 524 subjects agreed to enroll, 817 subjects refused enrollment. When subjects refused the study, they were asked why. The refusal responses were recorded in free from text by research coordinators.

#### Patient Selection at the Academic Hospital

10,124 subjects with recruitment enrollment data at the Academic hospital were identified and extracted from the REDCap system. A total of 4,164 subjects were excluded from the study for the following reasons: 3,032 subjects were ineligible based on enrollment criteria, 719 subjects were unable to be contacted, and 513 subjects did not complete the enrollment process within 5 days from receiving the prescription for the targeted medication. After exclusions, there were 5,860 eligible subjects successfully contacted and included in the final analysis (Figures 1B, 2B). 1199 subjects agreed to enroll, 4661 subjects refused enrollment. When subjects refused the study, they were asked why. The refusal responses were recorded in free form text by research coordinators.

#### Recruitment Variations and Limitations

While recruitment to the trial began in June 2015, various changes in sample collection methods, compensation for participation and EHR platform introduced potential bias in comparisons of subjects recruited prior to January 2017 at the Safety net hospital. Thus, for the purposes of this analysis, only subjects recruited after January 2017 at the Safety net hospital are included. Recruitment at the Academic hospital was approved December 2016 and enrollment began January 2017.

### Statistical Analysis

Statistical analyses were performed in the Statistical Package for the Social Sciences (SPSS; IBM Corp. Released 2017. IBM SPSS Statistics for Windows, Version 25.0. Armonk, NY, United States: IBM Corp). Descriptive statistics, i.e., mean, quartiles, frequencies, and percentages, were analyzed to describe baseline characteristics of the study participants. To test for differences in enrollment outcomes between races, MUA/P’s, and gender, a chi-squared test of independence was performed. A test of multi-collinearity was run to evaluate inter-correlations between the independent variables. Sensitivity analyses were performed between racial groups to identify the best strategy for grouping racial minority subjects with low enrollment. A forward conditional logistic regression method was used to select the best model in clinical trial eligible subjects. We built a logistic regression to estimate the effect of patient characteristics on successful enrollment into the INGENIOUS clinical trial.

Real data responses for the refusal category were transcribed, categorized, and analyzed. Frequencies and mean refusal responses were analyzed to determine reasons for not enrolling into the INGENIOUS Clinical Trial. Statistical analyses were performed independently at the Safety net and Academic hospital systems.

## Results

### Safety net Patient Demographics

A total of 1,341 subjects were included in the final analysis at the Safety net hospital. Female enrollment was 62.3%, male enrollment was 37.7%. Subjects living outside of MUA/P’s were 53.7%, while subjects living inside MUA/P’s were 45.3%. 36.8% self-identified as non-Hispanic White, 53.8% African-American, and 9.4% self-identified as Native Hawaiian or Pacific Islander, Indian or Alaska Native, Asian, more than one race, or refused to answer. The latter group was categorized into one racial category, termed, “other minority” (Table 1).

**TABLE 1:** Demographics of study participants.

Demographic characteristic^a^	Safety net hospital		Academic hospital	
	Number of subjects	Percent	Number of subjects	Percent
**Gender**				
Female	835	62.3	3863	65.9
Male	506	37.7	1997	34.1
**Age group (years)**				
18–38	299	22.3	1454	24.8
38–52	388	28.9	1302	22.2
52–63	426	31.8	1274	21.7
63–92	228	17.0	1830	31.3
**MUA/P**				
Yes	608	45.3	1714	29.3
No	720	53.7	4141	70.7
Missing	13		5	
**Race**				
Non-Hispanic White	494	36.8	4866	83.0
African-American/Black	721	53.8	795	13.6
Other minority	126	9.4	199	3.4
Total	1341	100.0	5860	100.0

*^*a*^Characteristics of study participants at the Safety net and Academic hospital systems. Abbreviations: MUA/P, Medically Underserved Area/Population.*

### Enrollment Outcomes-Safety net Hospital

Data responses were recorded for subjects, which either agreed or refused the INGENIOUS trial. A chi-square test of independence revealed there were no significant differences in enrollment patterns stratified by MUA/P (*p* = 0.90) and gender (*p* = 0.11), but significant differences in enrollment patterns by race (*p* < 0.001). Both race and age are significant covariates to predict enrollment at the Safety net hospital. African-Americans were less likely to refuse the study than non-Hispanic Whites (OR = 0.68, CI 0.53–0.86, and *p* = 0.002), while other minorities were more likely to refuse the study than non-Hispanic Whites (OR = 1.58, CI 1.02–2.45, and *p* = 0.04; Table 2). Additionally, the odds of refusing the study increased with age (OR = 1.02, CI, 1.02–1.03, *p* < 0.001; Table 2).

**TABLE 2:** Estimated odds ratios (OR) and 95% confidence intervals (CI) for enrollment refusal based on logistic regression analysis.

Predictor variables	Safety net (OR)	95% CI	Academic (OR)	95% CI
***Race***				
African-American/Black	0.68	0.53–0.86	0.64	0.53–0.76
Other minorities	1.58	1.02–2.45	1.28	0.88–1.87
***Gender***				
Female0/Males^1^	0.81	0.64–1.03	0.83	0.72–0.95
*Age*	1.02	1.02–1.03	1.02	1.02–1.02
***MUAP***				
No^0^ /Yes *MUA/P^1^*	1.05	0.81–1.32	0.81	0.70–0.94

*Abbreviations: CI, Confidence interval; MUA/P, Medically Underserved Areas/Populations. Reference groups: Living in MUA/P, males, and non-Hispanic Whites.*

### Patient Refusal Responses-Safety net Hospital

Refusal responses from open-ended questions were translated and categorized into similar barriers and the responses were stratified by race (Figure 2A; Supplementary Appendix A). All 19 categories are listed in Supplementary Appendix A. The most common reason both African-American and non-Hispanic White subjects refused to enroll was because they were not interested in the study. Other barriers to enrollment from the most frequent to the least included time or being too busy, the clinic or hospital site was too far or had a lack of transportation, and sick or had other issues. Trust was reported less frequently in both ethnic groups. Additionally, 40% of refusal responses were missing at the Safety net hospital because some subjects abruptly hung up on research coordinators after they answered the phone or either received the subjects’ voicemail. When subjects did give reasons for refusing the study, examples of soft refusals were, “Can’t get to the Safety net hospital,” “Just had surgery, please keep in mind for the future,” and “Can’t get to Safety net hospital that short of notice, keep in mind for future.” When subjects gave hard refusals, some examples were, “Take me off your list!,” “No, doesn’t trust my DNA on files anywhere,” “Hates the Safety net hospital, will not do a study,” or “Doesn’t have time for things like this; take my name off of your list.” (Figure 2A).

### Academic Hospital Patient Demographics

5,860 subjects were included in the final analysis at the Academic hospital. Female enrollment was 65.9%, male enrollment was 37.7%. Subjects living outside of MUA/P’s were 70.7% and within MUA/P’s were 29.3%. 83% self-identified as non-Hispanic White, 13.6% African-American or Black, and 3.4% Native Hawaiian or Pacific Islander, Indian or Alaska Native, Asian, more than one race or refused to answer. The latter group was categorized into one racial category, termed, “other minority” (Table 1).

### Enrollment Outcome-Academic Hospital

A chi-square test of independence revealed there were significant differences in enrollment patterns by race (*p* < 0.001), MUA/P (*p* = 0.01), and gender (*p* < 0.001). A forward logistic regression model selection process revealed race, age, MUA/P, and gender are significant covariates for enrollment into the INGENIOUS clinical trial at the Academic hospital. African-Americans were less likely to refuse the study than non-Hispanic-whites (OR = 0.64, CI 0.53–0.76, and *p* < 0.001; Table 2). Other minorities did not reach statistical significance in this analysis. Females were less likely to refuse the study than males (OR = 0.83, CI 0.72–0.95, and *p* = 0.01) and the odds of refusing the study increased with age (OR = 1.02, CI 1.02–1.03, and *p* < 0.001). Subjects not living in MUA/P’s were less likely to refuse the study than those living in MUA/P’s (OR = 0.81, CI 0.70–0.94, and *p* = 0.007; Table 2).

### Patient Refusal Responses-Academic Hospital

Refusal responses from open-ended questions were translated and categorized into similar barriers and the responses were stratified by race (Figure 2B; Supplementary Appendix A). All 19 categories are listed in Supplementary Appendix A. At the Academic hospital, the most common reason both African-American and non-Hispanic White subjects refused to enroll was due to a lack of interest in the study. Other barriers to enrollment include; time or being too busy, sick or had other issues, and being too far. Additionally, there were a number of non-Hispanic White respondents (*n* = 27) who reported they didn’t want their medicine changed and trust was an infrequent response as a barrier to enrollment for both African-Americans and non-Hispanic White respondents (Figure 2B). Additionally, 6% of refusal responses were missing at the Academic hospital because some subjects abruptly hung up on research coordinators after they answered the phone or either received the subjects’ voicemail. When subjects did give reasons for refusing the study, examples of soft refusals were, “Have too many doctor appointments at this time,” “Has too many things going on,” or “I want to stick with what my doctor said.” When subjects gave hard refusals, some examples were, “Not interested in hearing about a research study,” “I’m not taking the meds and not interested in the study,” or “I don’t have time and don’t think that this study would be beneficial to me.”

## Discussion

An analysis of enrollment outcomes into the INGENIOUS pharmacogenetic trial at the Safety net hospital and the Academic hospital in a diverse population was conducted. Historically, African-Americans have been underrepresented in research and clinical trials, with many researchers reporting mistrust as a major barrier to enrollment (Washington, 2006; Lincoln et al., 2018). Similarly, a recent study recorded viewpoints from African-Americans on receiving healthcare and found that some subjects felt they were being discriminated against, based on race and their health insurance coverage (Cuevas et al., 2017).

Due to reports of mistrust with research from minority populations, we hypothesized that minority populations enrolled into INGENIOUS would be less likely to enroll than non-Hispanic Whites. In our analyses, African-Americans at the Safety net and Academic hospital were less likely to refuse enrollment into this pharmacogenetic implementation trial than non-Hispanic Whites and support recent findings that suggest African-Americans are interested in participating in clinical trials when trust and support from providers exist (Robinson et al., 2016).

The Safety net hospital is a county, urban hospital, located in Indianapolis, Indiana and has 10 primary care sites in the Indianapolis area. In collaboration with the Indiana University School of Medicine, they provide a variety of health programs to their communities. One possible explanation for the unanticipated result which showed African-Americans were less likely to refuse enrollment into the INGENIOUS trial may be due to the high awareness of research studies conducted at this hospital. The Indiana Clinical and Translational Sciences Institute Research Network (ResNet) Program Manager was interviewed. Notably, ResNet clinical coordinators were involved in recruitment efforts at the Safety net and Academic hospitals. The ResNet Program Manager was asked why she thought African-Americans would agree to participate more than non-Hispanic Whites at the Safety net hospital. She replied, “At the Safety net hospital, African-American patients often expect to be asked to participate in research.” “In fact, subjects frequently participate in multiple independent research studies and know that research is a part of their healthcare.” Moreover, these subjects have had the opportunity to participate in over 500 active research studies since 2001^2^. Previous studies have also reported that awareness of research studies is important for improving clinical trial participation in African-Americans and medically underserved communities (Robinson et al., 2016) but may be undervalued in early research recruitment strategies. For prospective subjects to gain interest in pharmacogenetic trials, subjects need to know they exist. The Safety net hospital promotes research through online advertising, education, and collaborating with established research institutions, consequently exposing their subjects to various research opportunities.

Additionally, mostly African-American recruiters were involved in enrolling subjects in the INGENIOUS study at the Safety net hospital and may have influenced positive enrollment in our African-American population. A prior study suggests African-Americans and other minorities need health care professionals from their own cultural background to increase trust in the health care system (Kennedy et al., 2007). In addition, the clinical coordinators are a part of the Indiana CTSI ResNet and are highly skilled, with over 20 years of experience.

Cultural competency has also been reported to improve relationships with providers and minority subjects (Cuevas et al., 2017). A recent study conducted by Nielsen et al., 2019 implemented a cultural teaching program, which included 30 health professionals. The study demonstrated that improving cultural competencies enhance motivation in providers and strengthen relationships with minority subjects. Recruiters for the Safety net hospital are proficient in cultural competency and are experienced with recruiting diverse populations. Alternatively, Otado et al., 2015, reported a field-based strategy was the most impactful method for recruiting African-Americans and targeting a specific clinic was less effective.

We also evaluated the outcome of African-Americans being less likely to refuse the study than non-Hispanic Whites at the Academic hospital. Most subjects who enrolled at the Academic hospital were non-Hispanic White and were given an option to consent to the INGENIOUS trial by traveling to a site that was most convenient for them. Many responses from prospective subjects at the Academic hospital still reported that traveling was a barrier for participation. Although we cannot pinpoint exactly why prospective subjects who were non-Hispanic White had low enrollment, a fraction of participants reported not interested, being too busy/lack of time, being sick and the clinic or hospital being too far as top barriers to enrollment. While other explanations included work/other obligations and subjects not wanting their medication changed, which were more frequent in non-Hispanic White than African-American subjects (Figure 2B).

Individuals living in medically underserved areas also have many barriers to equal health care and research opportunities, compared to the general population (Tanner et al., 2015). Tanner et al., 2015 found that clinical trial researchers rarely engaged individuals in medically underserved communities to participate in clinical trials and experienced difficulty finding rural residents to participate in clinical trials. Additionally, in 2014, 27% of low-income adults in rural areas of Mississippi between the ages of 19–64, were reported to be uninsured and ranked the lowest in health, in the state of Mississippi (Wang et al., 2017). Thus, individuals living in medically underserved areas would greatly benefit from a targeted health care initiative and improved research efforts by sponsors. Our study revealed subjects not living in MUA/P’s were less likely to refuse the INGENIOUS trial than those subjects living in MUA/P’s at the Academic hospital (Table 2), which is what was expected. However, MUA/P status was not associated with enrollment outcomes at the Safety net hospital.

We observed similar results for age and enrollment into clinical trials as in previous studies (Sisk et al., 2008). The odds of refusing the study increased with age at the Safety net and Academic hospital. These findings support the notion that older subjects are less likely to enroll in clinical trials. Elderly subjects also experience health disparities, reducing biases associated with old age and raising cultural proficiency has been suggested to improve health care in this population (Periyakoil, 2019) and also may improve clinical trial enrollment.

Moreover, a recent report by the FDA, evaluated women’s participation into clinical trials worldwide and found that the United States had the highest enrollment of women (49.1%) compared to other countries; nevertheless, the majority of women who enrolled identified as white across various cardiovascular disease clinical trials (Ayuso et al., 2019). At the Academic hospital, women were less likely to refuse the study than men. Notably, increased enrollment reported for women in the United States, could be in part, due to the conscious effort of researchers to comply with the NIH policies on the inclusion of women (Taylor, 2008).

**FIGURE 3:**
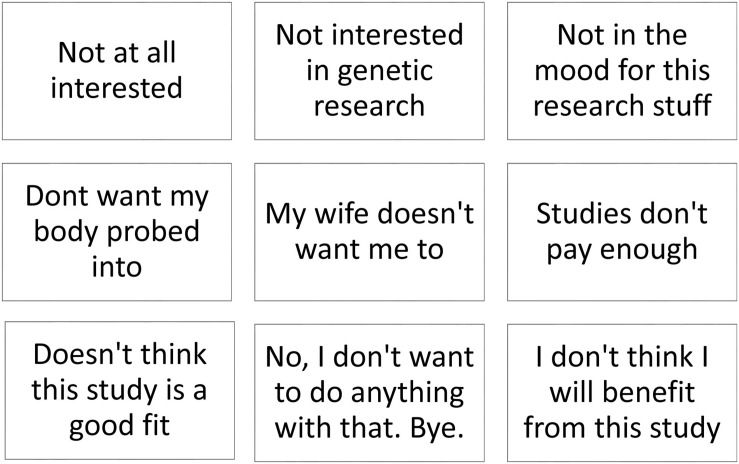
Prospective patients explanations for not being interested.

In contrast, understanding why potential subjects do not enroll in clinical trials is of equal importance. The top 4 responses for refusing to enroll were similar between African-Americans and non-Hispanic Whites at both hospital systems. Less frequently reported was trust, for both African-American and non-Hispanic Whites. We also attempted to understand the reason for the respondents’ lack of interest. Those explanations were highly variable, and included, “Don’t want my body probed into,” “Don’t pay enough,” “I don’t think I will benefit from this study,” “My wife doesn’t want me to,” and “Not in the mood for this research stuff.” (Figure 3). One drawback in the analysis was that the Safety net hospital data had 40% of the refusal responses missing and the Academic hospital had 6% of the refusal responses missing. In addition, with 19 different response categories, there were not enough data to make statistical comparisons between the responses for the different racial groups. However, a refusal response reported by African-American and non-Hispanic White subjects, “open to the study, but not at this time,” could be a unique opportunity to enhance research recruitment in future studies. These subjects clearly state they are interested in research, but perhaps had other commitments at the time of the INGENIOUS trial.

In summary, there were many barriers that prospective subjects reported for not enrolling into the INGENIOUS trial. Since lack of interest was the most frequent response in non-Hispanic White and African-American subjects, researchers should consider creative ways to promote interest in research to the general public before approaching perspective subjects by phone. Recent studies have demonstrated that implementing group seminars increase research participation (Shepp et al., 2018; Mayhew et al., 2020) and that telephone calls are less effective than in person recruitment (Chhatre et al., 2019).

Further, numerous studies have discussed strategies which may improve clinical trial participation for underserved populations. One of the common “themes” for improving enrollment for minority populations across genetic studies begin with building trust (Horowitz et al., 2019). Making trust a reality in underserved communities include; ensuring the research staff understands the challenges of the community, ensure a proportion of recruiters come from the community they are wanting to enroll, and establishing involvement from community stakeholders and subjects from minority populations in the beginning of the clinical trial design. Recruiters at the Safety net hospital reflected the population they were able to recruit, and likely influenced the positive enrollment at this hospital. Moving toward implementation of these strategies, which are likely to have a profound effect on successful enrollment are clearly the next steps.

Finally, personalized medicine approaches have improved drug exposure and response between racial/ethnic groups. Taking a personalized recruitment approach should increase clinical trial participation. Additionally, the implementation of the NIH inclusion policies by more trial sponsors should help to reduce healthcare disparities, by making the results relevant to more diverse racial/ethnic groups. This will undoubtedly make drugs safer and more efficacious in diverse populations.

## Data Availability Statement

The datasets generated for this study are available on request to the corresponding author.

## Ethics Statement

The studies involving human participants were reviewed and approved by Indiana University Institutional Review Board. The patients/participants provided their written informed consent to participate in this study.

## Author Contributions

ES-W, KL, YZ, AH, and TS: article preparation. ES-W, KL, YZ, AH, PD, and TS: conceptualization. CS, KL, PD, and TS: recruitment implementation. ES-W, YZ, AH, KL, and TS: data analysis. All authors contributed to the article and approved the submitted version.

## Conflict of Interest

The authors declare that the research was conducted in the absence of any commercial or financial relationships that could be construed as a potential conflict of interest.

## Footnotes

1 https://bhw.hrsa.gov/shortage-designation/types

2 https://www.eskenazihealth.edu/about/research
